# Supplementary material for: Skeletal muscle enhancer interactions identify genes controlling whole-body metabolism
Source: Nat Commun. 2020 Jun 1;11:2695. doi: 10.1038/s41467-020-16537-6 (PMC7264154; doi:10.1038/s41467-020-16537-6)
Supplement: Supplementary file 1 — Supplementary Information [file 41467_2020_16537_MOESM1_ESM.pdf]

Supplementary Information

Skeletal muscle enhancer interactions identify genes controlling whole-body metabolism

Williams et al.

Supplementary Figure 1

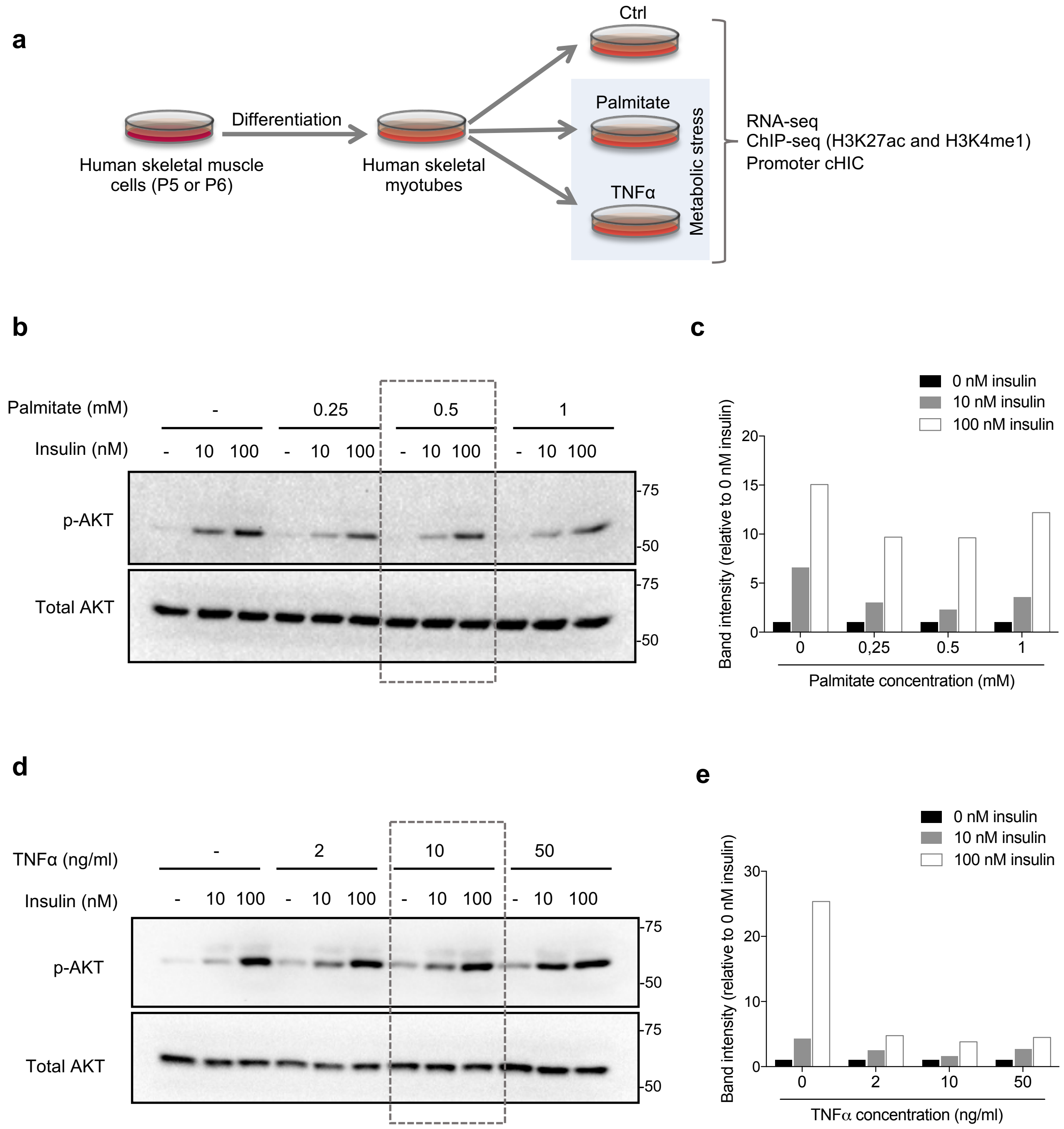

Supplementary Figure 1: Overview of human skeletal muscle cell culture experiments. a Human primary skeletal muscle cells of passage 5 or 6 were differentiated into myotubes and incubated with either palmitate for 48 h or TNFα for 24 h or left untreated as a control (ctrl). The cells were subsequently used for transcriptomic analysis (RNA-seq), enhancer mapping (ChIP-seq for H3K27ac and H3K4me1) and chromatin conformation assay (Promoter Capture Hi-C). **b, d** Western blot of p-AKT and total AKT from myotubes treated with different concentrations of palmitate (untreated, 0.25 mM, 0.5 mM or 1 mM) (b) or TNFα (untreated, 2 ng/ml, 10 ng/ml or 50 ng/ml) (d). The cells were stimulated with 10 or 100 nM insulin for 5 minutes before they were harvested and lysed. **c, e** Quantification of western blot bands from palmitate (c) or TNFα (e) stimulation.

Supplementary Figure 2

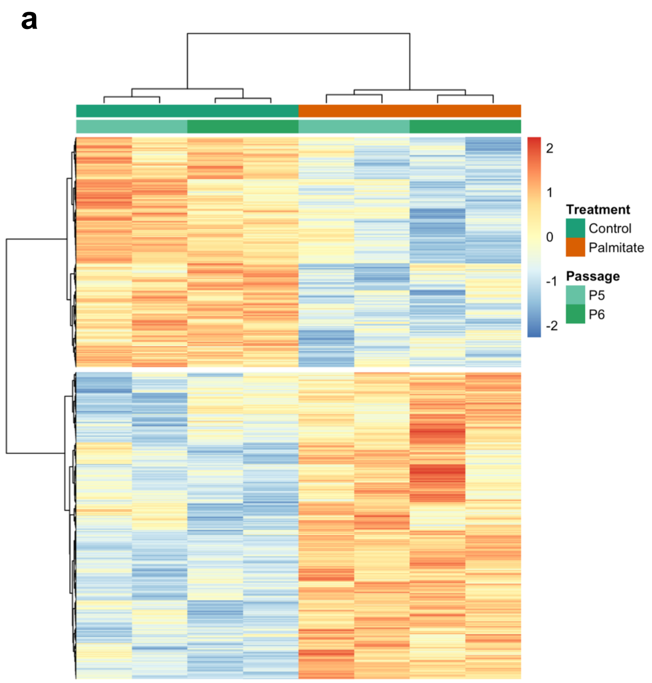

Supplementary Figure 2: Heatmap representation of RNA-seq data. Heatmap representation of RNA-seq data for palmitate (a) or TNFα (b) treatment. Groups (control, palmitate or TNFα) and cell culture passages (P5 or P6) are indicated in the top panel with the color codes showed in the legends.

Supplementary Figure 3

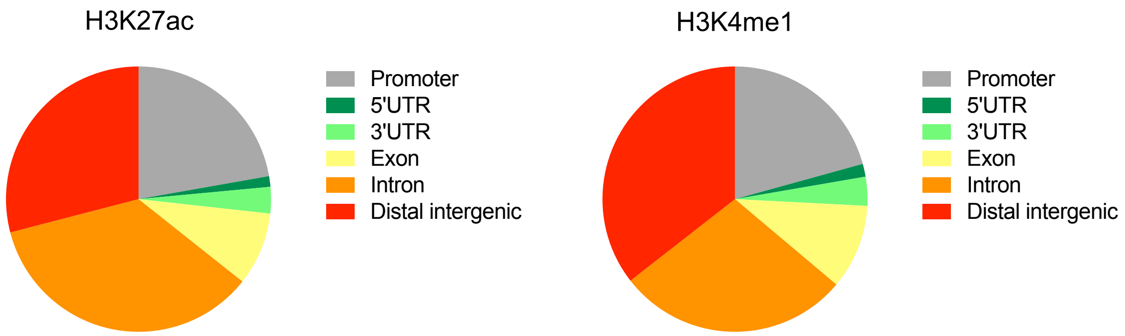

Supplementary Figure 3: Distribution of ChIP-seq peaks. Diagrams illustrating the distribution of H3K4me1 or H3K27ac ChIP-seq peaks. The peaks were annotated into promoters (+/- 3kb from TSS), 5'UTR, 3'UTR, exons, introns and distal intergenic.

Supplementary Figure 4

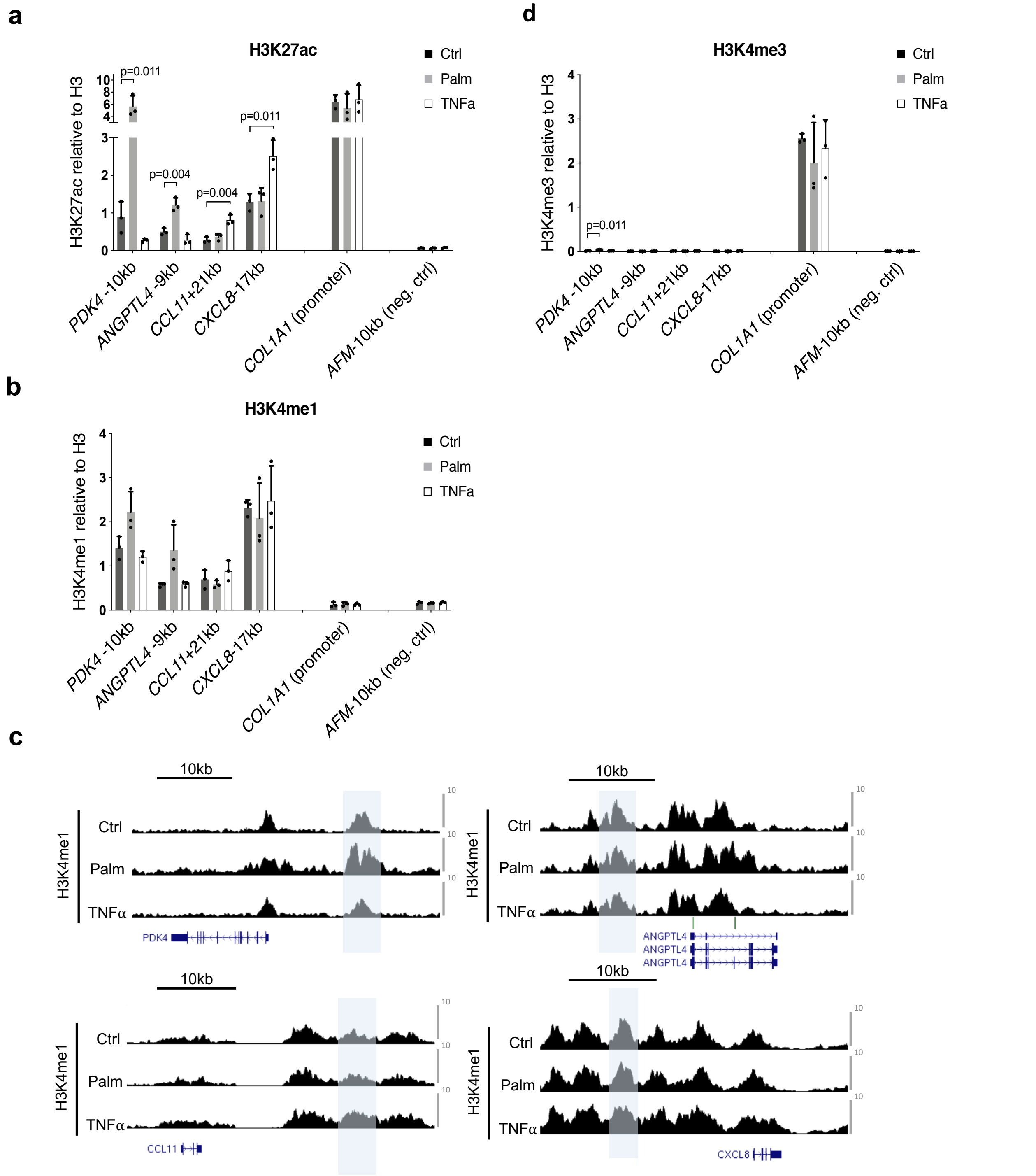

Supplementary Figure 4: Validation of ChIP-seq data by ChIP-qPCR. a-b H3K27ac (a) or H3K4me1 (b) ChIP-qPCR in control, palmitate or TNFα-treated human skeletal myotubes. qPCR was performed targeting *PDK4*-10kb, *ANGPTL4*-9kb, *CCL11*+21kb and *CCXL8*+6kb enhancer elements, as well as *COL1A1* promoter and *AFM*-10kb negative control region. Values are presented as mean ± S.D (n=3). Statistical test was performed by a two-tailed t-test and significant changes are indicated by p-values. All other changes were non-significant. **c** UCSC genome browser H3K4me1 tracks from control, palmitate or TNFα-treated cells. **d** H3K4me3 ChIP-qPCR in control, palmitate or TNFα-treated human skeletal myotubes. qPCR was performed targeting *PDK4*-10kb, *ANGPTL4*-9kb, *CCL11*+21kb and *CCXL8*+6kb enhancer elements, as well as *COL1A1* promoter and *AFM*-10kb negative control region. Values are presented as mean ± S.D (n=3). Statistical test was performed by a two-tailed t-test and significant changes are indicated by p-values. All other changes were non-significant.

Supplementary Figure 5

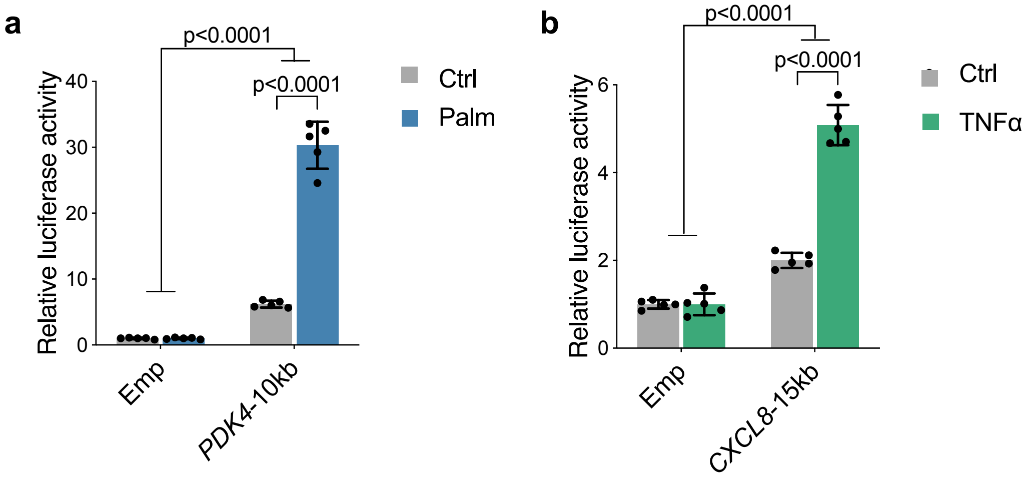

Supplementary Figure 5: Luciferase assays to validate enhancer activation. Relative luciferase activity in human skeletal muscle cells transfected with pGL4.23[luc2/minP] luciferase reporter plasmid, either empty or containing the *PDK4*-10kb (a) or *CXCL8*-17kb (b) enhancer. Transfected cells were left untreated (ctrl) or treated with palmitate (a) or TNFα (b). Values are represented as mean ± S.D (n=5) relative to the empty vector. Statistical test was performed by two-way anova.

.

Supplementary Figure 6

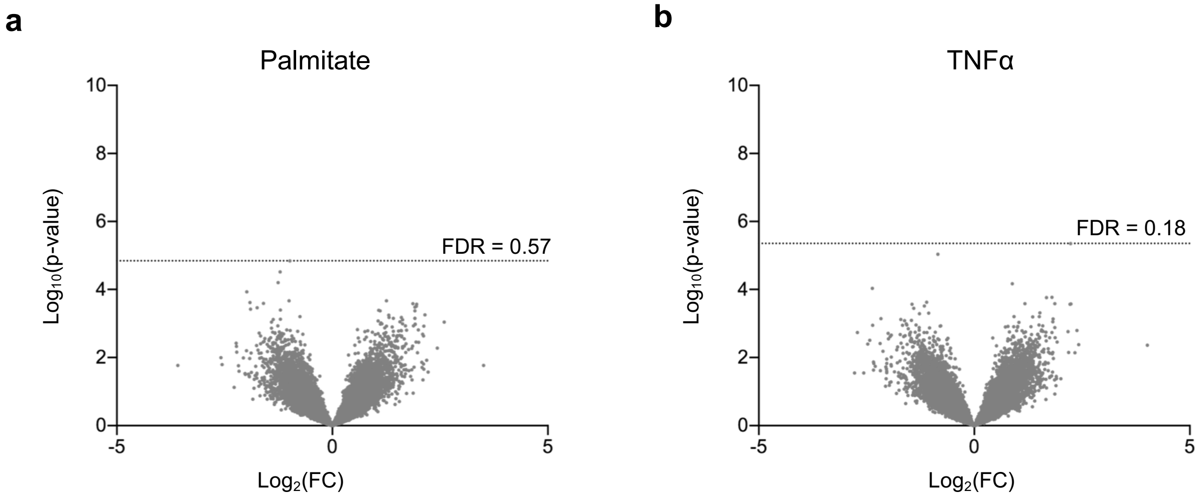

Supplementary Figure 6: Volcano plot presentation of Promoter Capture Hi-C interactions. **a, b** Volcano plot representation of the difference in Promoter Capture Hi-C interactions in skeletal muscle myotubes after treatment with palmitate (a) or TNFα (b). No interactions had an FDR-value below 0.57 for palmitate (a) or 0.18 for TNFα (b) treatment.

Supplementary Figure 7

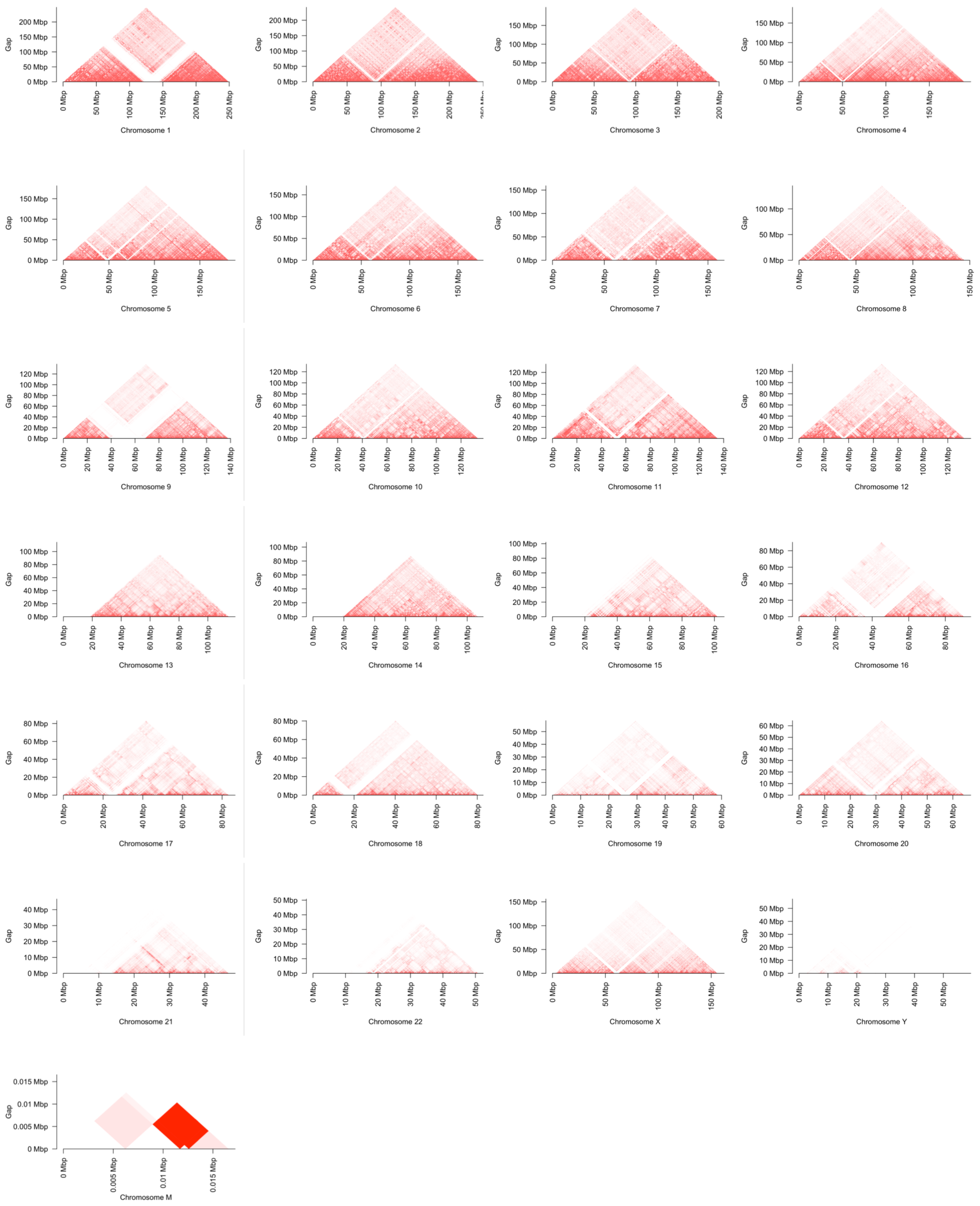

Supplementary Figure 7: Heatmap illustration of Promoter Capture Hi-C data. Data are presented for chromosome 1-22 and chromosome M, X and Y.

Supplementary Figure 8

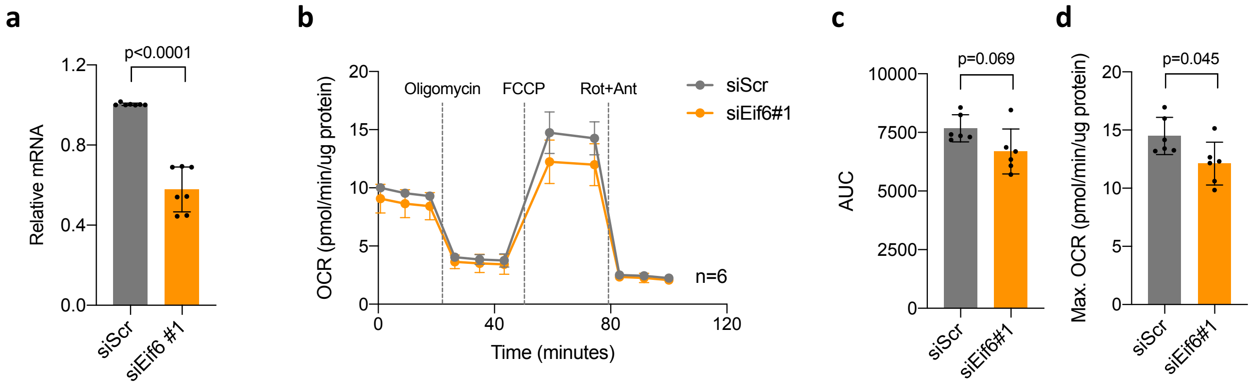

Supplementary Figure 8: Mitochondrial respiration after knockdown of *Eif6* using siEif6#1. a *Eif6* mRNA levels in siScr or siEif6#1 transfected C2C12 myoblasts. Expression data was normalized to housekeeping *Gapdh* expression levels. Values are represented as mean ± S.D (n=7), statistical test was performed by a paired two-tailed t-test. **b** Real-time measurements of oxygen consumption rates (OCR) by Seahorse Extracellular Flux Analyzer in siScr or siEif6#1 transfected C2C12 myoblasts. OCR was measured under basal conditions and after injection of oligomycin, FCCP, and antimycin A combined with rotenone at indicated time points. Values are represented as mean ± S.D (n=6). **c, d** OCR area under the curve (AUC) values (c) or mean OCR for the time points during FCCP-induced maximal respiration (d) for siScr or siEif6#1 transfected C2C12 cells. Values are represented as mean ± S.D (n=6), statistical test was performed by a paired two-tailed t-test.

Supplementary Figure 9

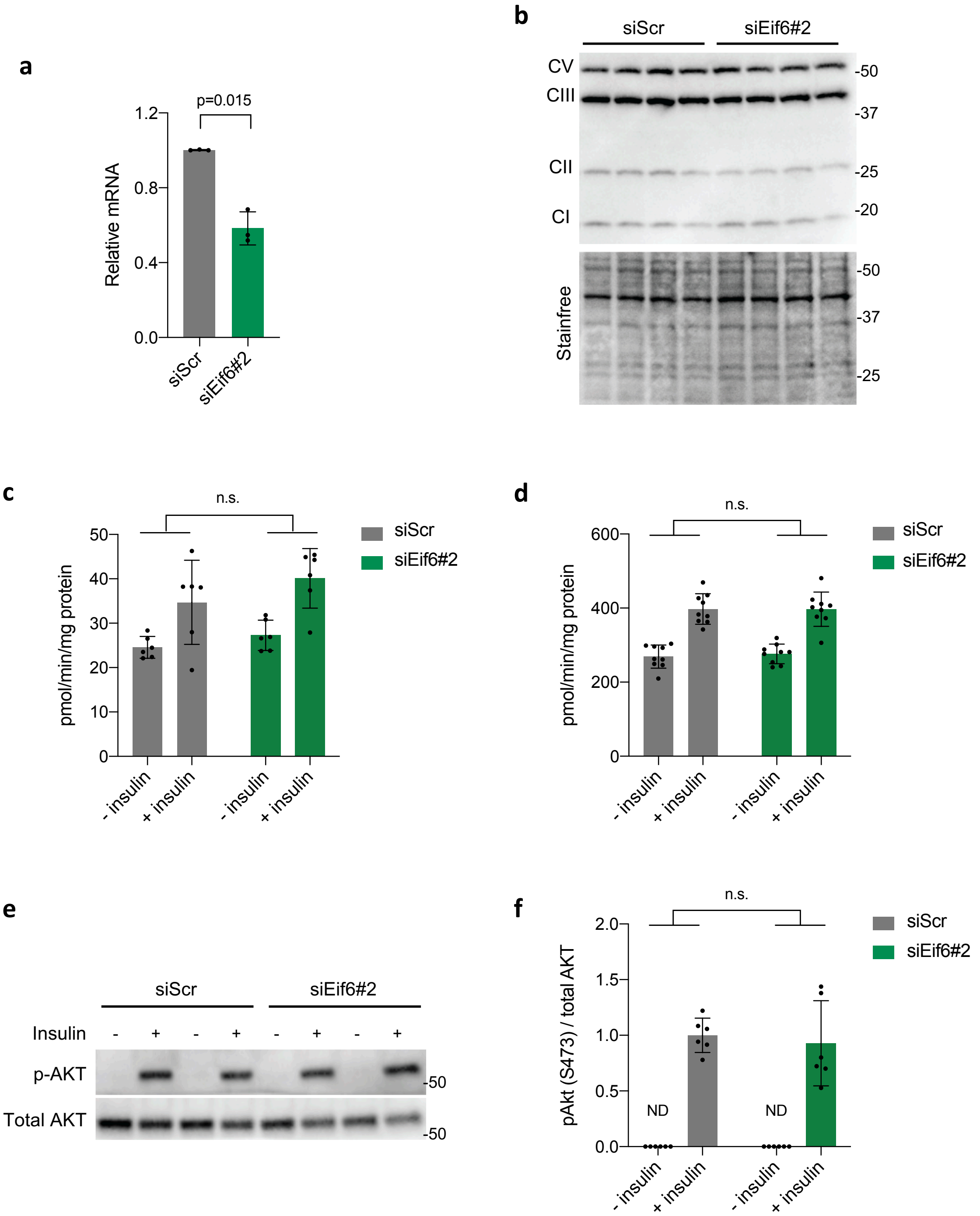

Supplementary Figure 9: Metabolic assays after knockdown of *Eif6* in muscle myotubes. a *Eif6* mRNA levels in siScr or siEif6#1 transfected C2C12 myotubes. Expression data was normalized to housekeeping *Gapdh* expression levels. Values are represented as mean ± S.D (n=3), statistical test was performed by a paired two-tailed t-test. **b** Representative western blot of the mitochondrial oxidative complexes V, III, II and I in siScr or siEif6#2 transfected C2C12 cells. **c, d** Glucose uptake ± 10 nM insulin (c) or glycogen synthesis ± 100 nM insulin (d) in siScr or siEif6#2 transfected C2C12 cells. Values are represented as mean ± S.D (n=6 for glucose uptake assay, n=9 for glycogen synthesis assay), statistical test was performed by a two-way ANOVA where there was a significant effect of insulin in both experiments but no effect of *Eif6* knockdown (n.s, p>0.05). **e, f** Representative western blot (e) and quantification (f) of AKT phosphorylation ± 10 nM insulin. Values are represented as mean ± S.D (n=6), statistical test was performed by a two-way ANOVA where there was a significant effect of insulin but no effect of *Eif6* knockdown (n.s, p>0.05).
