# Supplementary material for: Skeletal muscle enhancer interactions identify genes controlling whole-body metabolism
Source: Nat Commun. 2020 Jun 1;11:2695. doi: 10.1038/s41467-020-16537-6 (PMC7264154; doi:10.1038/s41467-020-16537-6)
Supplement: Supplementary file 3 — Description of Additional Supplementary Information [file 41467_2020_16537_MOESM3_ESM.pdf]

Description of Additional Supplementary Files

File Name: Supplementary Data 1
Description: RNA-seq data. LogFC indicates the fold change in gene expression levels between control and palmitate, or control and TNFα treatment.

File Name: Supplementary Data 2
Description: GO analysis using Camera of genes up- or downregulated by palmitate or TNF□ treatment. NGenes is the number of genes expressed.

File Name: Supplementary Data 3
Description: ChIP-seq data. Genomic position (hg38) of all identified enhancers regions (H3K4me1 and H3K27ac positive). LogFC indicates the fold change in H3K27ac levels between control and palmitate, or control and TNFα treatment.

File Name: Supplementary Data 4
Description: Promoter Capture Hi-C data. Data are listed as a .bedpe file.

File Name: Supplementary Data 5
Description: Complete list of enhancers overlapping one or more GWAS SNPs and interacting with a target gene promoter, and where the enhancer activity and target gene promoter is concurrently regulated by either palmitate or TNFα treatment. SNPs listed in bold represents primary GWAS SNPs, whereas SNPs in italic are SNPs that were found in high LD to a primary GWAS SNP.

File Name: Supplementary Data 6
Description: Summary of eQTL analysis with list of significant eGenes. The analyses were adjusted for age or for age and BMI.

File Name: Supplementary Data 7
Description: Overview of selected metabolic phenotypes in the BXD murine genetic reference population.

File Name: Supplementary Data 8
Description: Gene expression correlations in skeletal muscle with phenotypes in the BXD mouse strains. Spearman's rho, p-value and FDR value is indicated for each correlation.

File Name: Supplementary Data 9
Description: Gene expression correlations in adipose tissue with phenotypes in the BXD mouse strains. Spearman's rho, p-value and FDR value is indicated for each correlation.

File Name: Supplementary Data 10
Description: Gene expression correlations in liver tissue with phenotypes in the BXD mouse strains. Spearman's rho, p-value and FDR value is indicated for each correlation.

File Name: Supplementary Data 11
Description: Overview of all RNA-seq, ChIP-seq and promoter Promoter Capture Hi-C experiments.

File Name: Supplementary Data 12
Description: Complete list of primer sequences.
