# Supplementary material for: Skeletal muscle enhancer interactions identify genes controlling whole-body metabolism
Source: Nat Commun. 2020 Jun 1;11:2695. doi: 10.1038/s41467-020-16537-6 (PMC7264154; doi:10.1038/s41467-020-16537-6)
Supplement: Supplementary file 16 — Reporting Summary [file 41467_2020_16537_MOESM16_ESM.pdf]

nature research

Corresponding author(s): Romain Barrès

Last updated by author(s): Apr 9, 2020

Reporting Summary

Nature Research wishes to improve the reproducibility of the work that we publish. This form provides structure for consistency and transparency in reporting. For further information on Nature Research policies, see Authors & Referees and the Editorial Policy Checklist .

Statistics

For all statistical analyses, confirm that the following items are present in the figure legend, table legend, main text, or Methods section.

n/a	Confirmed	
☐	☒	The exact sample size (*n*) for each experimental group/condition, given as a discrete number and unit of measurement
☐	☒	A statement on whether measurements were taken from distinct samples or whether the same sample was measured repeatedly
☐	☒	The statistical test(s) used AND whether they are one- or two-sided<br>*Only common tests should be described solely by name; describe more complex techniques in the Methods section.*
☐	☒	A description of all covariates tested
☐	☒	A description of any assumptions or corrections, such as tests of normality and adjustment for multiple comparisons
☐	☒	A full description of the statistical parameters including central tendency (e.g. means) or other basic estimates (e.g. regression coefficient) AND variation (e.g. standard deviation) or associated estimates of uncertainty (e.g. confidence intervals)
☐	☒	For null hypothesis testing, the test statistic (e.g. *F*, *t*, *r*) with confidence intervals, effect sizes, degrees of freedom and *P* value noted<br>*Give P values as exact values whenever suitable.*
☒	☐	For Bayesian analysis, information on the choice of priors and Markov chain Monte Carlo settings
☒	☐	For hierarchical and complex designs, identification of the appropriate level for tests and full reporting of outcomes
☒	☐	Estimates of effect sizes (e.g. Cohen's *d*, Pearson's *r*), indicating how they were calculated

Our web collection on statistics for biologists contains articles on many of the points above.

Software and code

Policy information about availability of computer code

Data collection	No software used
Data analysis	RNA-seq reads were aligned with STAR v. 2.5.3.a, reads covering genes were counted using featureCounts v. 1.5.0-p1 and analysed for differential expression by edgeR v. 3.22.5 using recommended settings, and described more thoroughly in the paper. ChIP-seq reads were aligned with Subread v1.5.0. Duplicate reads were removed using Picard tools (http://broadinstitute.github.io/picard) and peaks was called using macs2 v. 2.1.1.20160309 and IDR v. 2.0.2 as described in the paper and in the section “Peak calling parameters”, reads covering ChIP peaks were counted using featureCounts v. 1.5.0-p1 and analysed for differential binding by edgeR v. 3.22.5 following the guidelines set in the paper (Lun, A.T. and G.K. Smyth, De novo detection of differentially bound regions for ChIP-seq data using peaks and windows: controlling error rates correctly. Nucleic Acid Res, 2014. 42(11): p. e95)<br>cHiC reads were processed by the HiCUP pipeline v. 0.6.1 and diffHic v. 1.12.1 was used for differential structure analysis and data handling.<br>Regional plots of GWAS data were generated using standalone LocusZoom v1.478.<br>Scans of western blots were analyzed using ImageLab software v6.0.1.<br>Genotypes were called on human skeletal muscle biopsies using the Genotyping module (version 1.9.4) of GenomeStudio software (version 2011.1, Illumina) and Illumina HumanCoreExome-12v1-0_B.egt cluster file.<br>All custom computer codes used for sequencing data analysis and figure generation is available at https://github.com/lars-work-sund/Skeletal-muscle-enhancer-interactions-identify-genes-controlling-human-metabolism.

For manuscripts utilizing custom algorithms or software that are central to the research but not yet described in published literature, software must be made available to editors/reviewers. We strongly encourage code deposition in a community repository (e.g. GitHub). See the Nature Research guidelines for submitting code & software for further information.

Data

Policy information about availability of data

All manuscripts must include a data availability statement. This statement should provide the following information, where applicable:

- Accession codes, unique identifiers, or web links for publicly available datasets
- A list of figures that have associated raw data
- A description of any restrictions on data availability

For analysis of RNA-seq data, reads were aligned to the hg38 GENCODE Comprehensive gene annotations [https://www.gencodegenes.org/]. H3K4me3 ChIP-seq peaks from human skeletal muscle myotubes were downloaded from Roadmap Epigenomics (sample E121) [https://egg2.wustl.edu/roadmap/data/byFileType/peaks/consolidated/narrowPeak/E121-H3K4me3.narrowPeak.gz]. Gene expression values from BXD cohorts were downloaded from the GEO data base [https://www.ncbi.nlm.nih.gov/geo/query/acc.cgi?acc=GSE60151] and [https://www.ncbi.nlm.nih.gov/geo/query/acc.cgi?acc=GSE60149], or from http://www.genenetwork.org/ [http://gn1.genenetwork.org/webqtl/main.py?FormID=sharinginfo&GN_AccessionId=779].

All novel sequencing data have been deposited in the NCBI Gene Expression Omnibus (GEO) and are accessible through GEO SuperSeries accession number GSE126102 [https://www.ncbi.nlm.nih.gov/geo/query/acc.cgi?acc=GSE126102]. RNA-seq data from GSE126101 [https://www.ncbi.nlm.nih.gov/geo/query/acc.cgi?acc=GSE126101] have been used to generate Figure 1A-I, Figure 2J and K, Figure 3E-H, Figure 6C, and Supplemental Figure 2. ChIP-seq data from GSE126099 [https://www.ncbi.nlm.nih.gov/geo/query/acc.cgi?acc=GSE126099] have been used to generate Figure 2A-I, Figure 3E-H, Figure 6B, Supplemental Figure 3, and Supplemental Figure 4. Promoter Capture Hi-C data from GSE126100 [https://www.ncbi.nlm.nih.gov/geo/query/acc.cgi?acc=GSE126100] have been used to generate Figure 3A-H, Figure 6A, Supplemental Figure 6, and Supplemental Figure 7.

Field-specific reporting

Please select the one below that is the best fit for your research. If you are not sure, read the appropriate sections before making your selection.

[x] Life sciences [ ] Behavioural & social sciences [ ] Ecological, evolutionary & environmental sciences

For a reference copy of the document with all sections, see nature.com/documents/nr-reporting-summary-flat.pdf

Life sciences study design

All studies must disclose on these points even when the disclosure is negative.

Sample size	No sample-size predetermination was performed in this study. However, by using n=4 biological replicates for ChIP-seq in cultured human skeletal muscle cells (control cells or cells treated with palmitate or TNFα) from two different cell passages, we were able to detect 1632 and 18026 enhancers, respectively, with altered H3K27ac levels using a FDR<0.01. As a comparison, a recent study investigating changes in H3K27ac in hepatocytes of high fat diet-fed mice uses six animals in each group and are able to detect 883 sites with changes in H3K27ac with FDR<0.1 (Siersbæk et al. High fat diet-induced changes of mouse hepatic transcription and enhancer activity can be reversed by subsequent weight loss. Sci Rep. 2017 Jan 10;7:40220).<br>We therefore estimate that the sample size used in this study was sufficient.
Data exclusions	No data was excluded
Replication	All attempts at replication was successful
Randomization	Randomization was performed whenever applicable and relevant. For example when processing samples for ChIP-seq the samples were fixated, washed and de-crosslinked in random orders.
Blinding	Blinding was not was not performed in this study since we did not find relevance for this

Reporting for specific materials, systems and methods

We require information from authors about some types of materials, experimental systems and methods used in many studies. Here, indicate whether each material, system or method listed is relevant to your study. If you are not sure if a list item applies to your research, read the appropriate section before selecting a response.

Materials & experimental systems

n/a	Involved in the study
[ ]	[x] Antibodies
[ ]	[x] Eukaryotic cell lines
[x]	[ ] Palaeontology
[x]	[ ] Animals and other organisms
[ ]	[x] Human research participants
[x]	[ ] Clinical data

Methods

n/a	Involved in the study
[ ]	[x] ChIP-seq
[x]	[ ] Flow cytometry
[x]	[ ] MRI-based neuroimaging

Antibodies

Antibodies used	H3K4me1 from Abcam (Ab8895). Diluted 1:200 for ChIP-seq.<br>H3K27ac from Abcam (Ab4729). Diluted 1:200 for ChIP-seq.<br>H3K4me3 from Cell Signaling Technology (CST-9751). Diluted 1:200 for ChIP-seq.<br>H3 from Abcam (Ab1791). Diluted 1:200 for ChIP-seq.<br>AKT from Cell Signaling Technology (CST-9272). Diluted 1:1000 for western blotting.<br>Phospho-Ser-AKT from Cell Signaling Technology (CST-9271). Diluted 1:1000 for western blotting.<br>Total OXPHOS Rodent WB Antibody Cocktail (Ab110413). Diluted 1:5000 for western blotting.<br>Goat anti-rabbit IgG horseradish peroxidase conjugate (Bio-Rad 170-6515). Diluted 1:10,000 for western blotting.<br>Goat anti-mouse IgG horseradish peroxidase conjugate (Bio-Rad 170-6516). Diluted 1:10,000 for western blotting.
Validation	H3K4me1 antibody used for ChIP-seq: Polyclonal antibody from Abcam with catalog number Ab8895. According to Abcam homepage all batches of Ab8895 are specific for mono-methylated Lysine 4 of histone H3 and does not recognise di- or tri-methyl Lysine 4 nor methylation at Lysine 9. Furthermore, this antibody has been used for ChIP-seq in several other studies including: Emmett MJ et al. Histone deacetylase 3 prepares brown adipose tissue for acute thermogenic challenge. Nature 546:544-548 (2017) and Sabò A et al. Selective transcriptional regulation by Myc in cellular growth control and lymphomagenesis. Nature 511:488-92 (2014).<br><br>H3K27ac antibody used for ChIP-seq: Polyclonal antibody from Abcam with catalog number Ab4729. According to Abcam homepage all batches of ab4729 are tested using peptide arrays and show less than 30% cross reactivity with both Histone H3 acetyl K9 and unmodified Histone H3 peptides. Furthermore, this antibody has been used for ChIP-seq in several other studies including: Emmett MJ et al. Histone deacetylase 3 prepares brown adipose tissue for acute thermogenic challenge. Nature 546:544-548 (2017) and Shu S et al. Response and resistance to BET bromodomain inhibitors in triple-negative breast cancer. Nature 529(7586):413-417 (2016).<br><br>H3K4me3 antibody used for ChIP-qPCR: Polyclonal antibody from Cell Signaling Technology with catalog number CST-9751. According to CST this antibody shows some cross-reactivity with histone H3 that is di-methylated on Lys4, but does not cross-react with non-methylated or mono-methylated histone H3 Lys4. In addition, the antibody does not cross-react with methylated histone H3 Lys9, Lys27, Lys36 or methylated histone H4 Lys20.<br><br>H3 antibody used for ChIP-qPCR: Polyclonal antibody from Abcam with catalog number Ab1791.<br><br>AKT antibody used for WB: Polyclonal antibody from Cell Signaling Technology with catalog number CST-9272. According to CST this antibody detects endogenous levels of total Akt1, Akt2 and Akt3 proteins. The antibody does not cross-react with related kinases.<br><br>Phospho-Ser473 AKT used for WB: Polyclonal antibody from Cell Signaling Technology with catalog number CST-9271. According to CST this antibody also recognizes Akt2 and Akt3 when phosphorylated at the corresponding residues. It does not recognize Akt phosphorylated at other sites, nor does it recognize phosphorylated forms of related kinases such as PKC or p70 S6 kinase.<br><br>Total OXPHOS Rodent WB Antibody Cocktail: Antibody cocktail from Abcam with catalog number Ab110413. The OXPHOS cocktail contains 5 mouse mAbs, one each against CI subunit NDUFB8 (ab110242), CII-30kDa (ab14714), CIII-Core protein 2 (ab14745) CIV subunit I (ab14705) and CV alpha subunit (ab14748) as an optimized premixed cocktail.

Eukaryotic cell lines

Policy information about cell lines

Cell line source(s)	Human Skeletal Muscle Cells, CC-2561, was obtained from Lonza<br>Murine C2C12 skeletal muscle myoblast cell line, CRL-1772, was obtained from ATCC.
Authentication	The human CC-2561 and the murine CRL-1772 C2C12 cell lines have not been authenticated, however both cell lines were passaged for a maximum of 12 passages after purchase.
Mycoplasma contamination	The human CC-2561 and the murine CRL-1772 C2C12 cell lines were tested negative for mycoplasma contamination.
Commonly misidentified lines<br>(See ICLAC register)	No commonly misidentified cell lines were used

Human research participants

Policy information about studies involving human research participants

Population characteristics	The study participants were Danish white men selected from the Danish draft boards records. 1,930 obese individuals and 3,601 randomly selected individuals for the population-representative control group were invited to participate in the study. In total 557 individuals volunteered to participate. From a subset of these, 145 individual skeletal muscle biopsies were taken. The participants were healthy by self-report and under 65 years of age at the time of examination.
Recruitment	See above
Ethics oversight	The study was approved by the Ethics Committee from the Capital Region of Denmark and informed consent was obtained from all participants in accordance with the Declaration of Helsinki II

Note that full information on the approval of the study protocol must also be provided in the manuscript.

ChIP-seq

Data deposition

- [x] Confirm that both raw and final processed data have been deposited in a public database such as GEO.
- [x] Confirm that you have deposited or provided access to graph files (e.g. BED files) for the called peaks.

Data access links
May remain private before publication.

The data has been deposited in GEO with Series accession number GSE126102. Use the following secure token to get access: urwraysgzrqhfqv

Files in database submission

Input.fastq.gz
P5_Ctrl_Rep1_K27ac.fastq.gz
P5_Ctrl_Rep1_K4me1.fastq.gz
P5_Ctrl_Rep2_K27ac.fastq.gz
P5_Ctrl_Rep2_K4me1.fastq.gz
P5_Palm_Rep1_K27ac.fastq.gz
P5_Palm_Rep1_K4me1.fastq.gz
P5_Palm_Rep2_K27ac.fastq.gz
P5_Palm_Rep2_K4me1.fastq.gz
P5_TNFa_Rep1_K27ac.fastq.gz
P5_TNFa_Rep1_K4me1.fastq.gz
P5_TNFa_Rep2_K27ac.fastq.gz
P5_TNFa_Rep2_K4me1.fastq.gz
P6_Ctrl_Rep1_K27ac.fastq.gz
P6_Ctrl_Rep1_K4me1.fastq.gz
P6_Ctrl_Rep2_K27ac.fastq.gz
P6_Ctrl_Rep2_K4me1.fastq.gz
P6_Palm_Rep1_K27ac.fastq.gz
P6_Palm_Rep1_K4me1.fastq.gz
P6_Palm_Rep2_K27ac.fastq.gz
P6_Palm_Rep2_K4me1.fastq.gz
P6_TNFa_Rep1_K27ac.fastq.gz
P6_TNFa_Rep1_K4me1.fastq.gz
P6_TNFa_Rep2_K27ac.fastq.gz
P6_TNFa_Rep2_K4me1.fastq.gz
k27ac_consensus.bed.gz
k27ac_counts.csv.gz
k4me1_consensus.bed.gz
k4me1_counts.csv.gz
Ctrl_K27ac.bw
Ctrl_K4me1.bw
Palm_K27ac.bw
Palm_K4me1.bw
TNFa_K27ac.bw
TNFa_K4me1.bw

Genome browser session
(e.g. UCSC)

We have not been able to generate an link to a genome browser session, since we do not have access to a FTP site. We have instead uploaded bigWig (.bw) files of ChIP-seq data to GEO database.

Methodology

Replicates	H3K4me1 and H3K27ac ChIP-seq of primary human skeletal muscle cells (CC-2561 from Lonza) was performed in four biological replicates where we used cells from two different passages (passage 5 and passage 6, two replicates from each passage).
Sequencing depth	A full overview of all ChIP-seq experiments including the number of PCR cycles used during each library preparation, the total number of reads, and the number of uniquely mapped reads is included as Supplemental Table S7.
Antibodies	The H3K4me1 antibody used was a polyclonal antibody from Abcam with catalog number Ab8895. According to Abcam homepage all batches of Ab8895 are specific for mono-methylated Lysine 4 of histone H3 and does not recognise di- or tri-methyl Lysine 4 nor methylation at Lysine 9. Furthermore, this antibody has been used for ChIP-seq in several other studies including: Emmett MJ et al. Histone deacetylase 3 prepares brown adipose tissue for acute thermogenic challenge. Nature 546:544-548 (2017) and Sabò A et al. Selective transcriptional regulation by Myc in cellular growth control and lymphomagenesis. Nature 511:488-92 (2014).<br><br>The H3K27ac antibody used was a polyclonal from Abcam with catalog number Ab4729. According to Abcam homepage all batches of ab4729 are tested using peptide arrays and show less than 30% cross reactivity with both Histone H3 acetyl K9 and unmodified Histone H3 peptides. Furthermore, this antibody has been used for ChIP-seq in several other studies including: Emmett MJ et al. Histone deacetylase 3 prepares brown adipose tissue for acute thermogenic challenge. Nature 546:544-548 (2017) and Shu S et al. Response and resistance to BET bromodomain inhibitors in triple-negative breast cancer. Nature 529(7586):413-417 (2016).
Peak calling parameters	Reads were aligned using the Subread aligner (Liao, Y., G.K. Smyth, and W. Shi, The Subread aligner: fast, accurate and scalable read mapping by seed-and-vote. Nucleic Acids Res, 2013. 41(10): p. e108.) against a full index of the main chromosomes of the hg38 reference genome, as genomic DNA and keeping only uniquely mapped reads. Duplicate reads were removed using Picard tools (http://broadinstitute.github.io/picard). Peaks were called using MACS2 (Zhang, Y., et al., Model-based analysis of ChIP-Seq (MACS). Genome Biol, 2008. 9(9): p. R137.) using standard parameters except for the --keep-dup=all flag (duplicates were removed prior to MACS analysis) with input control and a p-value cutoff of 0.05. For individual samples, this was purely to assess the homogeneity of the samples and highlight poor samples (less than 200.000 peaks, or failure to estimate fragment length), no samples were discarded.
Data quality	For each histone modification, a consensus peak set was generated as follows: all samples were pooled, the pooled reads were shuffled and split in two (pseudo replicates). Peaks were called as above on each of these three samples (pool and two pseudo replicates). Finally, a consensus peak list was generated using the irreproducible discovery rate software ("Measuring reproducibility of high-throughput experiments" (2011), Annals of Applied Statistics, Vol. 5, No. 3, 1752-1779, by Li, Brown, Huang, and Bickel) with the pseudo replicate peak lists as input and the pooled peak list as oracle peak list. A lenient threshold of 0.05 was used
Software	Subread v. 1.5.0<br>IDR v. 2.0.2<br>macs2 v. 2.1.1.20160309<br>edgeR v. 3.22.5<br>samtools v. 1.3.1<br>Picard v. 1.128<br>STAR v. 2.5.3a<br>featureCounts v. 1.5.0-p1<br>HiCUP v. 0.6.1<br>diffHic v. 1.12.1
